# HIDM: Hybrid Intrusion Detection Model for Industry 4.0 Networks Using an Optimized CNN-LSTM with Transfer Learning

**DOI:** 10.3390/s23187856

**Published:** 2023-09-13

**Authors:** Umesh Kumar Lilhore, Poongodi Manoharan, Sarita Simaiya, Roobaea Alroobaea, Majed Alsafyani, Abdullah M. Baqasah, Surjeet Dalal, Ashish Sharma, Kaamran Raahemifar

**Affiliations:** 1 Department of Computer Science and Engineering, Chandigarh University, Gharuan, Mohali 140413, India; 2 College of Science and Engineering, Hamad Bin Khalifa University, Qatar Foundation, Doha P.O Box 5825, Qatar; 3 Apex Institute of Technology (CSE), Chandigarh University, Gharuan, Mohali 140413, India; 4 Department of Computer Science, College of Computers and Information Technology, Taif University, P.O. Box 11099, Taif 21944, Saudi Arabia; 5 Department of Information Technology, College of Computers and Information Technology, Taif University, P.O. Box 11099, Taif 21974, Saudi Arabia; 6 Amity School of Engineering and Technology, Amity University, Gurugram 122412, India; 7 Department of Computer Engineering and Applications, GLA University, Mathura 281406, India; 8 Data Science and Artificial Intelligence Program, College of Information Sciences and Technology, Penn State University, State College, PA 16801, USA; 9 School of Optometry and Vision Science, Faculty of Science, University of Waterloo, 200 University, Waterloo, ON N2L3G1, Canada; 10 Faculty of Engineering, University of Waterloo, 200 University Ave W., Waterloo, ON N2L3G1, Canada

**Keywords:** Industry 4.0, cyber security, deep learning, optimized CNN, LSTM, transfer learning, GWO

## Abstract

Industrial automation systems are undergoing a revolutionary change with the use of Internet-connected operating equipment and the adoption of cutting-edge advanced technology such as AI, IoT, cloud computing, and deep learning within business organizations. These innovative and additional solutions are facilitating Industry 4.0. However, the emergence of these technological advances and the quality solutions that they enable will also introduce unique security challenges whose consequence needs to be identified. This research presents a hybrid intrusion detection model (HIDM) that uses OCNN-LSTM and transfer learning (TL) for Industry 4.0. The proposed model utilizes an optimized CNN by using enhanced parameters of the CNN via the grey wolf optimizer (GWO) method, which fine-tunes the CNN parameters and helps to improve the model’s prediction accuracy. The transfer learning model helps to train the model, and it transfers the knowledge to the OCNN-LSTM model. The TL method enhances the training process, acquiring the necessary knowledge from the OCNN-LSTM model and utilizing it in each next cycle, which helps to improve detection accuracy. To measure the performance of the proposed model, we conducted a multi-class classification analysis on various online industrial IDS datasets, i.e., ToN-IoT and UNW-NB15. We have conducted two experiments for these two datasets, and various performance-measuring parameters, i.e., precision, F-measure, recall, accuracy, and detection rate, were calculated for the OCNN-LSTM model with and without TL and also for the CNN and LSTM models. For the ToN-IoT dataset, the OCNN-LSTM with TL model achieved a precision of 92.7%; for the UNW-NB15 dataset, the precision was 94.25%, which is higher than OCNN-LSTM without TL.

## 1. Introduction

Industry 4.0 promotes digitizing conventional industrial production and working practices through intelligent technologies that facilitate machine-to-machine connectivity. In industrial automation, all of the production, job processing, and operation mechanisms are automated using advanced IoT strategies and instruments [1]. An IoT technique in industrial automation is called “IIoT” (Industrial Internet of Things). This industrial automation principle also takes on machine-to-machine (M-M) and human-to-machine (H-M) communications.

Recently, IoT technology has been increasingly accepted by numerous industrial sectors, which include medical services, the energy sector, healthcare, the manufacturing and supply chain, and home appliance makers [2]. In IR 4.0 architectures, the key techniques that are widely used are virtualization, automation, ML and DL methods, artificial intelligence techniques, and IoT. In IR 4.0, these latest advancements are blended into various sectors of smart manufacturing and communication. In all of these sectors, data are available on cloud servers or remote platforms, which may cause intrusion attacks; thus, information security is always demanding in IR 4.0. IR 4.0 utilizes devices and networks for communication and data transmission, which increases the security risk. Any smart device connected to a public communication network can offer an easy entrance for intruders and cybercriminals to accomplish malicious activities, which can lead to huge data losses for an organization and can financially affect them.

The rapid utilization of IoT in industrial assignments has enhanced industry working processes and workers’ livelihoods. With the help of cutting-edge innovations such as data transmission techniques, software solutions, remote monitoring, networking, sensors, and embedded technology, IoT technology transforms a primary standard physical object into an intelligent entity.

**Figure 1:**
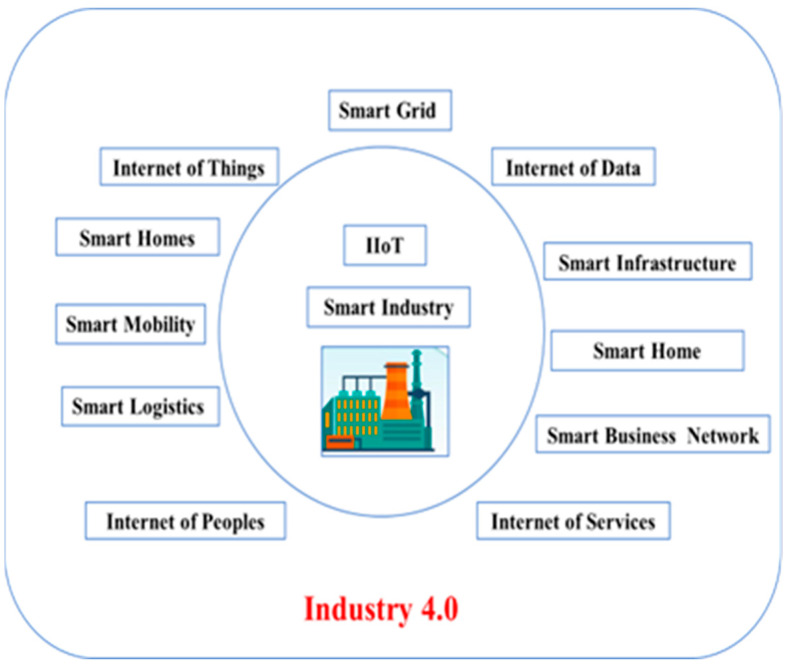
IIoT key components under the IR 4.0 environment.

In the IR 4.0 environment, the data flow among private and public networks for various purposes, increasing the demand for secure IoT network communications [3]. Figure 1 presents the significant components of IIoT under Industry 4.0;it mainly includes the Internet of Things (IoT), Internet of Data (IoD), Internet of People (IoP), and Internet of Services (IoS) technologies.

In IIoT communication, many appliances can directly accept and process any communication data in the IIoT network without checking for the security. These issues make the devices more sensitive to confidentiality and security risks and may endanger their IIoT solutions and automation implementations [4].

Due to the rapid evolution of IIoT, several security issues have arisen; to deal with these issues in the existing research, various conventional AI methods have beenutilized, which have been unable to resolve them properly. In the existing research, deep learning methods were found to be accurate and had numerous benefits that could be implemented to handle Industry IoT cyber threats [5]. These attacks have significant safety-related repercussions and adversely influence the system’s functions. Therefore, intrusion attacks are one of the most crucial challenges in Industry 4.0. In the IR 4.0 ecosystem, every day, various cyberattacks occur, which may result in monetary and non-monetary losses. Therefore, it is critical to recognize these threats and enhance and maintain IR 4.0 network security [6].

This research uses deep learning algorithms to develop a security model for detecting intrusions in IIoT networks under Industry 4.0 environments. The motivation and critical contribution of this study includes the following:

- This research presents a hybrid intrusion detection model using an optimized CNN and transfer learning for cyber threat detection in Industry 4.0.
- In the proposed model, the parameters of the CNN model were optimized using the GWO method, which fine-tunes the CNN parameters, i.e., pooling size, kernel size, number of filters, number of epochs, and batch size, which helps to enhance the model’s prediction accuracy.
- Using multi-class classification, the proposed hybrid model, existing OCNN-LSTM [1], and the CNN and LSTM model were tested on two popular IIoT datasets, ToN-IoT and UNW-NB15.
- An experimental analysis was performed using various performance-measuring parameters, i.e., precision, F-measure, recall, accuracy, and detection rate; a comparison analysis was performed betweenthe existing OCNN-LSTM model and the proposed HIDM model.
- In the experimental results, the proposed HIDM model achieved a precision of 92.7% for the ToN-IoT dataset and 94.25% for the UNW-NB15 dataset.

The complete article is divided into several sections, which are organized as follows. Section 2 covers the literature review of the existing research and cybersecurity risk factors in Industry 4.0. Section 3 covers the materials and methods related to the present study, the processes of the proposed HIDM architecture, and the dataset description. Section 4 covers the experimental results and discussion. Section 5 covers the conclusion and future work.

## 2. Related Work

This section analyzes deep and machinelearning-based research for ID detection in IR 4.0. Furthermore, it covers existing methods and IDS design patterns for identifying security breaches in IIoT networks.

The principle of IIoT has popped up due to the application of IoT in the production industry for unique and memorable activities predicated on emerging technology advances. An IIoT system comprises an actuator, sensor, control system communication channels, integration interface, cutting-edge surveillance systems, automotive communications networks, and intelligent appliances [7]. Everything in IIoT can be monitored via anonline platform. Using Industry 4.0 in several industries has enhanced the capabilities of many sectors, including equipment performance standards, consumer safety, monitoring solutions, and supply chain systems, and its use has substantially increased labor production efficiency. Moreover, the IIoT network enables a network area to become fully interactive by authorizing various applications, transmission modules, and quality assurance [8].

A framework for preventing cyberattacks on IIoT equipment using CNN was discussed in [9]. An auto-encoder cleaned up the statistics in the two models, ensuring a decent projection. A deep convolutional neural network was used to empower intrusion detection and classification. The suggested model was evaluated using the dataset, ISOT, and X-IIoTID. Another research that used the X-IIoTID data source introduced a highly reliable approach fordata communication;the presented system relies on deep learning. Deep neural networks were used to put the developed framework into the experiment. Based on the findings, a maximum accuracy value was accomplished [10].

A new approach for limiting and maintaining security streams to organizational and outsourced production line equipment, in addition to perimeter protection, is a necessary first step in securing industrial production as it adopts Industry 4.0 practices [11]. In research using UNSW-NB15 and KDD99 datasets, a comparative procedure was performedusing different strategies, including CNN and ANN. The research analyzed the data using performance measures such as false alarm frequency and precision value [12].

In the KDD-99 dataset, anticipation maximization had a precision of 792.6% and a false alarm percentage of 22.9%. An ID for IIoTwas suggested in another research study. A genetic algorithm (GA) was used to specify characteristics throughout the proposed model. The GA fitness value was accomplished using the random forest method [13]. A classification process achieved an under-the-curve area of 0.91 and a validation accuracy of 86.69% when using a UNSW-NB15 industry IDS dataset [14]. The GA-RM model that produced these outcomes had 16 characteristics. An IoT-based ID system utilizing deep learning for industry IoT networks was addressed in [13]. The feature extraction throughout this proposed model was conductedusing an optimization technique. The support vector machine (SVM) model was chosen for the intrusion classifier. The particle swarm optimization (PSO) method used in this study was predicated on a “Light Gradient Boosting” (Light-GB) technique [15].

Ref. [16] proposed a variation of a long short-term memory-based system for IIoTtechnology in the manufacturing industry. The attributes were rebuilt throughout the proposed IDS. After that, a selection criterion was operated among all of the completely new attributes. The features were determined using an auto-encoder method;this method extracts features from massive, complicated sample data [17]. Throughout this application, the investigators have used UNSW-NB15 samples. Another research developed an ensemble learning-based IDS for Industry 4.0 networks [18]. A principal responsive function was used for feature reduction in this method. The attributes of the responsive correlation coefficients that were decided upon were addressed to the transfer learning for categorization. The proposed framework was tested throughout the NSL-KDD and UNSW-NB15 datasets [19].

Furthermore, the multi-class classifiers method was carried out across both samples. A deep learning-based IDS system for IIoT was discussed in [20]. The suggested IDS was tested using samples from the X-IIoT-ID and ToN-IoT industrial datasets. The precision, F1-score, and accuracy were measured for the proposed model and compared with the existing ANN model [21]. Table 1 presents a comparative analysis of the existing IDS research.

## 3. Materials and Methods

This section presents the materials and methods related to the research.

### 3.1. Dataset Details

This research utilized the UNW-NB15 and ToN-IoT industrial datasets. The detailed descriptions of these datasets are as follows.

#### 3.1.1. UNW-NB15

This dataset was released in 2015 by the Central Australian Core for Cyber Lab and is widely used by academic researchers. The researchers utilized raw data packets created by the IXIA perfect combination system for the UNSW-NB15 time series. This dataset is ideal for IIoT attack analyses [22]. Table 2 presents the description of the UNW-NB15 dataset, and Table 3 presents the complete details of the UNW-NB15 dataset. The dataset is divided into an 80:20 ratio for training and testing.

**Figure 2:**
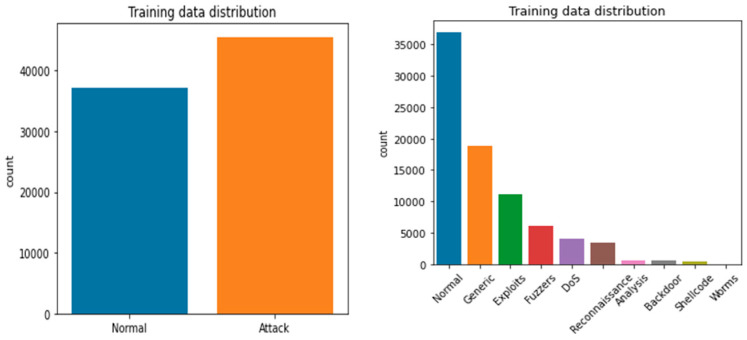
Visual representation of the UNW-NB15 training dataset.

**Figure 3:**
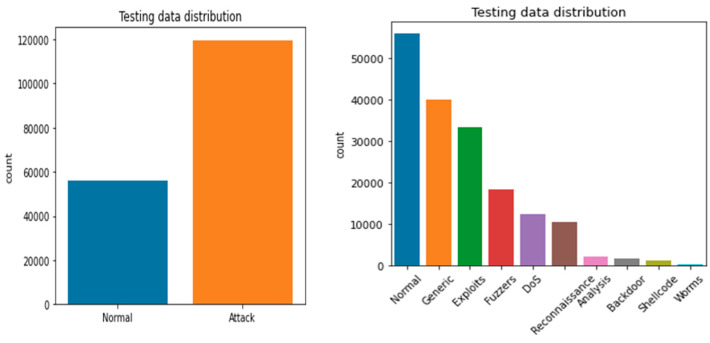
Visual representation of UNW-NB15 testing dataset.

The UNB dataset contains normal, back-door, worms, reconnaissance, fuzzers, DoS, shell code, exploits, and generic data attributes [23]. The dataset includes 38,500 normal records, 950 back-doors, 174 worms, 978 reconnaissance, 7500 fuzzers, 4800 DoS, 978 shellcode, 12,440 exploits, and 18,554 generic. The detailed description of UNW-NB15 is represented in Figure 2 and Figure 3. Figure 2 presents the training and testing dataset, and Figure 3 presents the visual representation of the training and testing dataset.

#### 3.1.2. ToN-IoT

This dataset is designed to accumulate and examine mashed datasets from the Industrial IoT, including diversified data, such as sensor data from the IoT, system logs, and network traffic details. A feasible network creates the IoT for industry [24]. The ToN-IoT dataset connects several virtual servers, cloud nodes, and physical devices. It includes assessing the precision and effectiveness of numerous machine intelligence information security implementations.

This dataset contains industrial cyber attacks. The tests used various aspects, including Win-7, Win-10, network samples, transmission detail, and IoT training models and their respective attacks. TheToN-IoT dataset contains normal, denialofservice, back-door, distributed denialofservice, MITM, injections, ransomware, scanning, cross-site scripting, and password records [25].

The ToN-IoT dataset contains attack classes and the numbers of instances for each class, wherethe details are as follows: normal, 79,638; denialofservice, 33,753; back-door, 50,811; distributed denialofservice, 61,650; MITM, 105; injections, 45,265; ransomware, 7280; scanning, 71,401; cross-site scripting, 21,089; and password, 17,185. Table 3 presents a detailed description of the ToN-IoT dataset.

### 3.2. Proposed HIDM

This research presents a hybrid intrusion detection model for an IIoT network for I4.0. The proposed model utilizes an optimized CNN with transfer learning. The proposed model uses the quality of transfer learning in the training and operates it in the CNN. A TL algorithm takes a framework trained on a massive volume of data and applies its expertise to a relatively small database. We freeze the network’s initial CNN architecture for classification tasks and only train the final few layers that help to make a final prediction [26].

**Figure 4:**
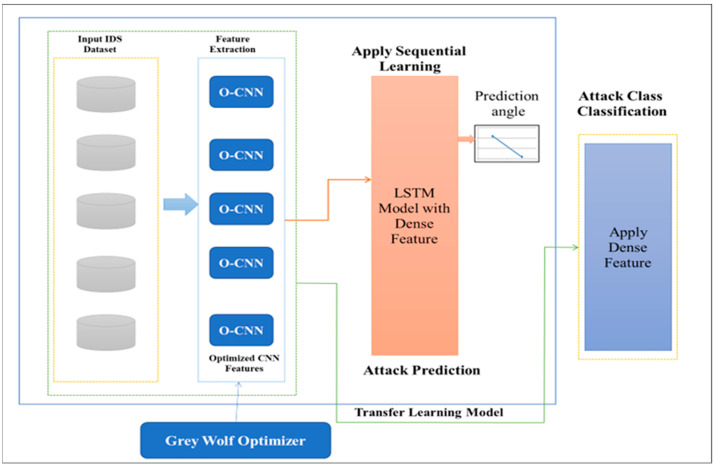
Architecture of the proposed model.

Figure 4 presents the architecture of the proposed HIDM model. The complete architecture is divided into three sections: optimized CNN, LSTM, and transfer learning. To enhance the CNN performance, we utilize fine-tuned parameters, i.e., learning rate, epoch, and weight; these all help optimize the CNN. The increased number of epochs in CNN helps to enhance the accuracy. The proposed model utilizes a GWO method [27] to optimize CNN hyperparameters.

#### 3.2.1. Optimized CNN usingthe GWO Method

In the proposed model, the existing CNN method is optimized by using the GWO method (Algorithm 1). GWO is used to tune the hyperparameters of the CNN method. Hyperparameters heavily influence the precision and efficiency of CNN. The selection of the network’s hyperparameters seems critical and is determined by the activity where the CNN is utilized. The hyperparameter for the CNN learning model includes “Batch size, Number of epochs, Pooling size, Number of Filters, and Kernel size.” The learning algorithm controls the gradient descent method’s performance, and the speed determines how much updating the earlier weights affects the updating of subsequent weights [28].

A frequency of epochs controls how frequently the learning algorithm updates the connection weights following the training sample. The overfitting problem in the connection is resolved via the regularization process as discussed in Algorithm 1. To handle all of these variables, adjusting these hyperparameters is essential to assist the network in producing the most detailed findings [29].
**Algorithm 1:** GWO method for hyperparameter optimization for CNN**Input:** input population, Batch size B_c_, Agents Ag, dimension Dn, Hyper-parameters H1, H2, H3, and H4, n is the number of iterations, Hyperparameter function H_fd_, Initialization of population P1, P2 and P3 
**Output:** Optimized hyper-parameter Select a data sample for training from batch input 
**For** the number of optimization, n  **For** search desire agent                 Determine the optimized fitness function                 Select the best search agents A1, A2, and A3.                 Update the position of each agent by
(1)Pos_in=∑k=0nnkA1kaAnn−k
                       where n is the number of iterations and k position change 
**end for**
 Update P_1_, P_2_ and P_3_
               End        Sigmoid function (H_1_, H_2_, H_3_,H_4_, H_fd_) End

#### 3.2.2. OCNN-LSTM with Transfer Learning

An OCNN-LSTM blended module was used in this proposed HIDM model. After the pre-processing, the CNN needed information from various sources, i.e., the local patterns in data again. Because the convolution kernels proceed in a fixed direction to dynamically extract the unobserved characteristics in the flow directions, the one-dimensional CNN performs very well in time-series network traffic workloads.

The CNN-LSTM framework includes CNN layers for extracting features on training datasets with long short-term memory for sequence learning. CNN-LSTMs are created to solve IDS prediction of Industry 4.0intrusiondetectionproblems and to generate explanations from observation sequences in IIoT datasets. We perform the CNN architecture with each input and send the outcome to the LSTM in a continuous sampling interval.

Transfer learning, as applied to the proposed HIDS, is just moving the weights of the CNN-LSTM model that have been trained over the IDS dataset to some other. Several data processing activities have benefited from the TL approach. This happens because the feature sequences that are primarily obtained by the bottom levels of the CNN architectures usually generate heavy features that can be applied to various applications, whereas only the top layers learn vital characteristics for a given dataset. As a result, the bottom levels of convolutional networks can be immediately applied to a broad variety activity. Fine-tuning can be employed in the TL procedure of learning algorithms to enhance the efficiency of TL [30,31,32,33,34,35,36].

### 3.3. Performance-Measuring Parameters

We utilize five broadly utilized specifications analyzing the model’s performance: precision (*PC*), accuracy (*AC*), true positive rate (*TPR*), false positive rate (*FPR*), and *F*1-score [30,31].

(2) $$AC=\frac{\left(TP+TN\right)}{\left(TP+TN+FP+FN\right)}*100$$

(3) $$TPR=\frac{TP}{\left(TP+FN\right)}*100$$

(4) $$FPR=\frac{FP}{\left(FP+TN\right)}*100$$

(5) $$Precision=\frac{TP}{\left(TP+FP\right)}*100$$

(6) $$Recall=\frac{TP}{\left(TP+FN\right)}*100$$

(7) $$F1=2*\frac{\left(Precision*Recall\right)}{\left(Precision+Recall\right)}*100$$

## 4. Experimental Results

This section covers the experimental scenario, setup, parameters, and result comparisons.

### 4.1. Dataset and Data Pre-Processing

This research utilizedthe popular ToN-IoT and UNW-NB15 Industrial IoT datasets. These data samples include numerous problems, such as missing values, excessive features, redundancy, and behavioral duplicates, whichdegrade model performance. We used class weights to counterbalance the data and improve the classifier performance. From each attack subclass category and its general behavior and vulnerability condition, a ranked column is incorporated within the ToN-IoT samples. The dataset was presented in the CSV file format. This dataset mainly contains nine key attack classes. The attack samples in UNSWB-15 were separated among nine classes in a CSV format called “normal class” and “attack class.” We identified and eliminated records with incomplete data, i.e., the NaNclass.

Categorical data in the dataset must be transformed into numerical values because DL techniques only work on numeric data. The Label Encoder class from the sklearn package was utilized for this conversion. Each attribute value was assigned a numerical value. The dataset contains properties measured at different levels in the specimen. The sample data must be expressed in a consistent specific metric to accurately assess these specimens employing DL techniques. With this aim, by using the Standard Scaler class within the sklearn public library, the results of the observations in the dataset were normalized well with the Z-Score normalization technique with just a mean average of zero as well as a normal deviation of one.

Additionally, we added regular vectors towards the “NaNclass,” changed the “NaNclass” variables to zero, and eliminated fixed attributes that served no purpose (e.g., dsport, srcip, dstip, and sport). When applying a feature-based strategy, independent of the duration of the attack, it is critical to “know” the properties of all previous windows. Each window is transmitted in a single packet to detect whether previous transmissions triggered an attack more on the current datagram.

### 4.2. Experimental Details

The proposed HIDS model and existing *OCNN-LSTM* model [1], CNN, and LSTM models were implemented using Python programming and tested on two popular online datasets, ToN-IoT and UNW-NB15. The performance of the proposed method was tested in two experiments. The first experiment was performed on the ToN-IoT dataset, and the second was performed on the UNW-NB15 dataset.

#### 4.2.1. Experiment1 for TON-IoT Dataset

The ToN-IoT dataset discussed in Section 3 includes diverse statistics collectedfrom various platforms utilized in Industrial IoT; it includesWin-7, Win-10, network samples, transmission details, andIoT.

In this experiment, the proposed HIDM model (OCNN-LSTM with transfer learning) and the existing OCNN-LSTM [1] model were used in a multi-class context to measure their effectiveness. The dataset was divided into 70:30 ratios for training and testing. The tests used various aspects, includingWin-7, Win-10, network samples, transmission detail, andIoT training models. The results were calculated for epoch 50 and epoch 100. Table 4 presents the outcome of the proposed model for the TON-IoT dataset.

**Figure 5:**
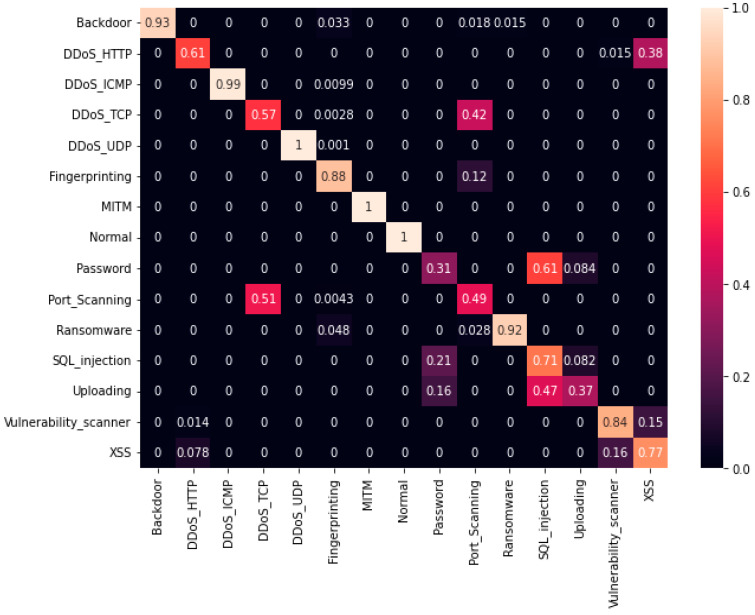
Confusion matrix for the proposed HIDM model for the ToN-IoT dataset.

**Figure 6:**
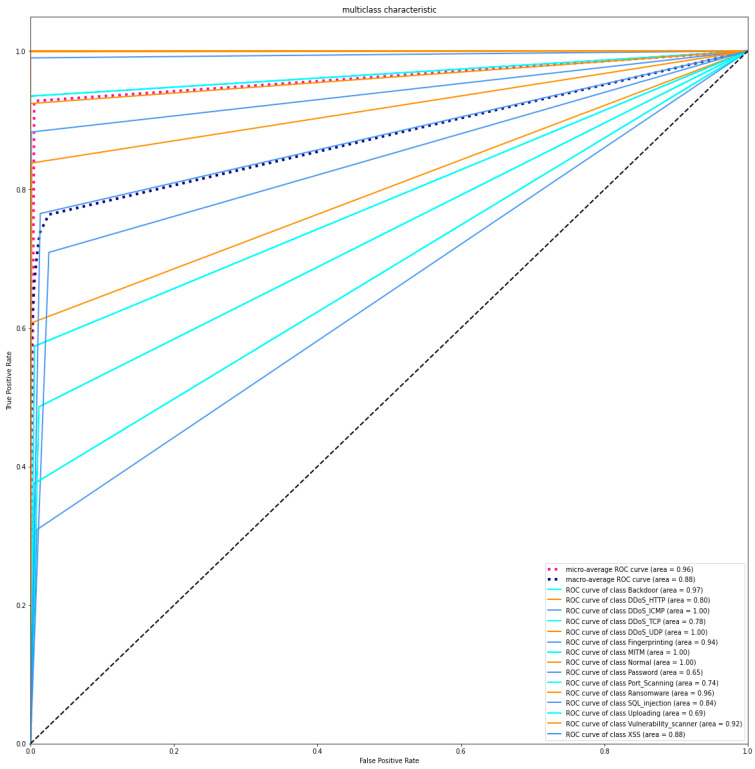
ROC Curve (TPR vs. FPR) of the proposed model for the ToN-IoT dataset.

**Figure 7:**
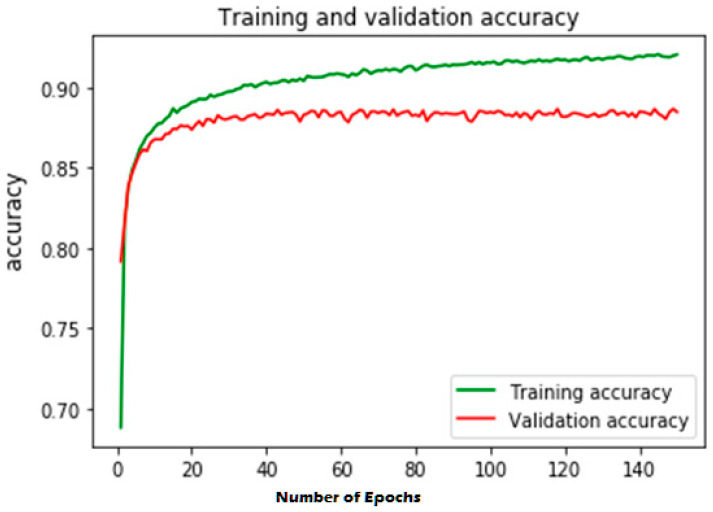
Training and validation accuracy of the proposed model for the ToN-IoT dataset.

**Figure 8:**
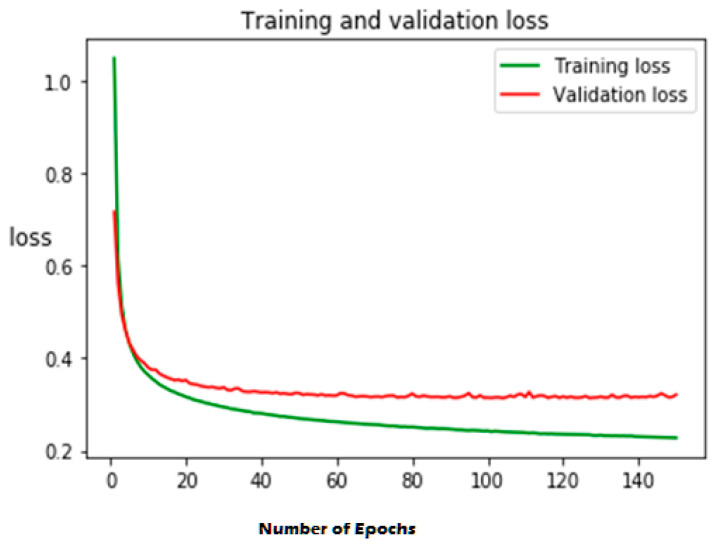
Training and validation loss of the proposed model for the ToN-IoT dataset.

The first experiment calculates various simulation results for OCNN-LSTM [1] and the proposed OCNN-LSTM with transfer learning. Figure 5, Figure 6, Figure 7 and Figure 8 present the simulation results for Experiment 1 of the proposed model for the ToN-IoT dataset. Figure 5 presents the simulation results of the confusion matrix for the proposed HIDM model. Figure 6 presents the results for the ROC Curve (TPR vs. FPR), Figure 7 presents the simulation results of the training and validation accuracy, and Figure 8 presents the simulation results of the training and validation loss of the proposed model for the ToN-IoT dataset.

Table 5 presents the simulation results of the experiment of the proposed model and the existing CNN-LSTM, simple CNN and LSTM models for the ToN-IoT dataset. The existing CNN-LSTM [1] model achieved a precision of 89.1%, recall of 49.6%, accuracy of 87.3%, and F1-score of 48.6%. The existing CNN model acquired a precision of 81.1%, recall of 48.1%, accuracy of 73.1%, and F1-score of 42.1%, and the similar existing LSTM model achieved a precision of 85.1%, recall of 47.1%, accuracy of 78.1%, and F1-score of 44.1%. The existing CNN and LSTM models have less accuracy.

We applied the transfer learning method with an optimized CNN and LSTM in the proposed model. The optimized CNN fine-tuned the hyperparameters, and the TL method enhanced the prediction accuracy by using knowledge transformation. The proposed model achieved a precision of 92.7%, recall of 52.39%, accuracy of 94.4%, and F1-score of 56.6%. This experimental analysis has proven that after applying the TL method, the performance of the CNN-LSTM model is slightly increased. The proposed model presents better results.

#### 4.2.2. Experiment2 for UNW-NB15 Dataset

The UNW-NB-15 dataset discussed in the sub-sections of Section 3 includes diverse statistics collected from various platforms that are utilized in Industrial IoT. The dataset contains ten classes of multiple attacks and forty-three features.

In this experiment, the proposed HIDM model (OCNN-LSTM with transfer learning) and existing OCNN-LSTM [1] model were used in a multi-class context to measure its effectiveness. The dataset was divided into 80:20 ratios for training and testing. The results were calculated for epoch 50 and epoch 100. Table 5 presents the outcome of the proposed model for the UNW-NB-15 dataset.

**Figure 9:**
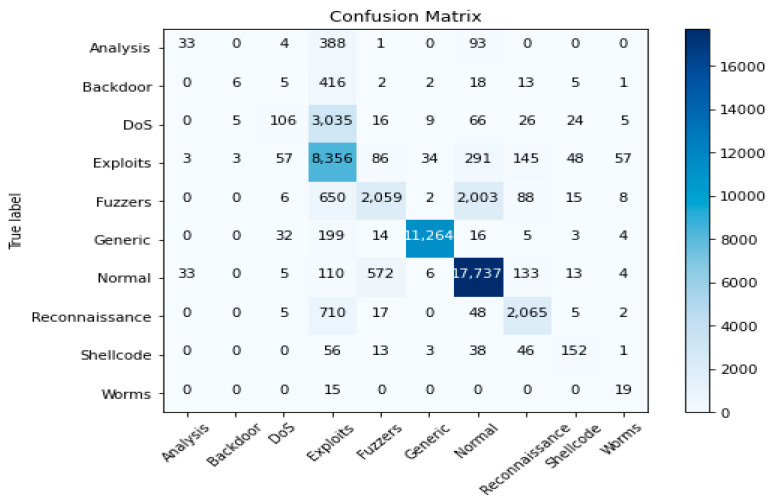
Confusion matrix of the proposed model for UNW-NB15.

**Figure 10:**
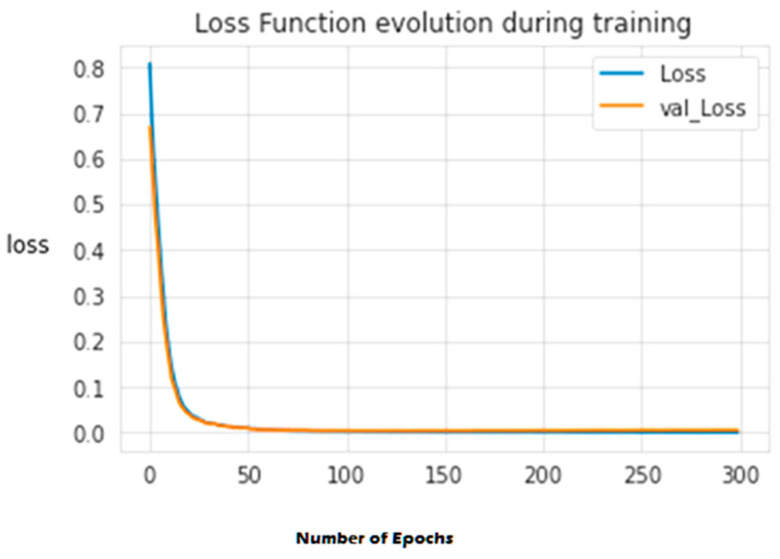
Loss function evolution during training of the proposed model for UNW-NB15.

**Figure 11:**
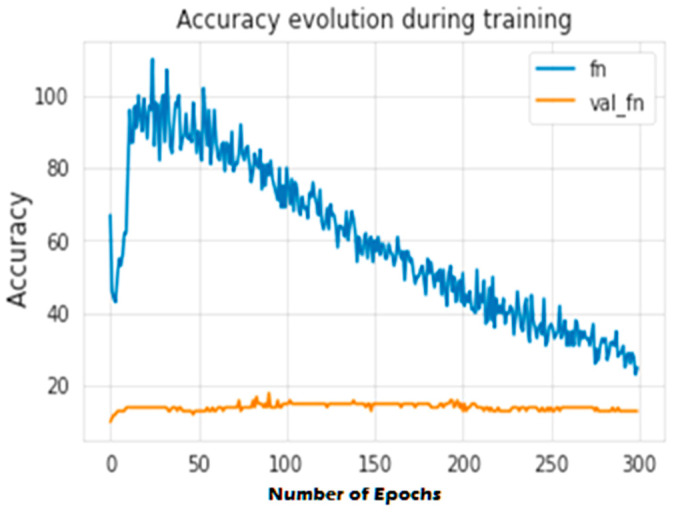
Accuracy evolution during training of the proposed model for UNW-NB15.

**Figure 12:**
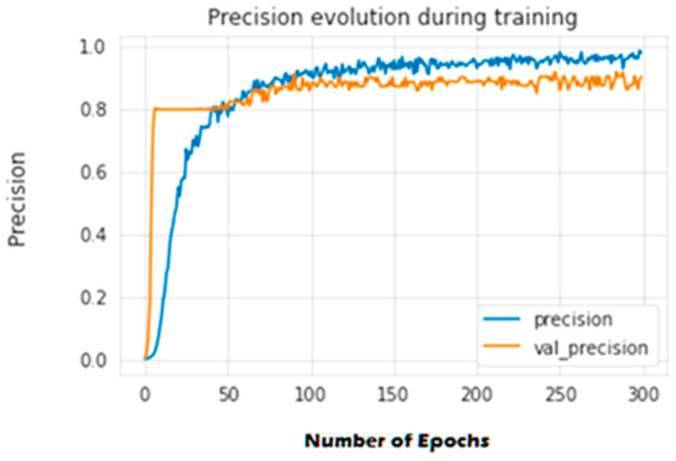
Precision evolution during training of the proposed model for UNW-NB15.

**Figure 13:**
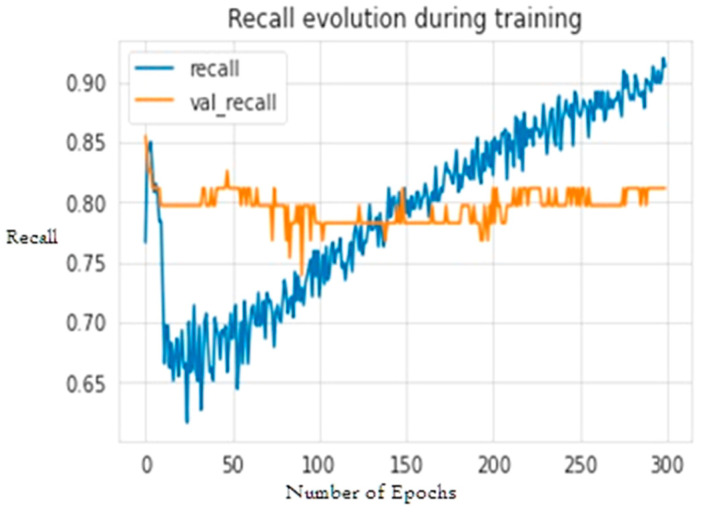
Recall curve for training of the proposed model for UNW-NB15.

**Figure 14:**
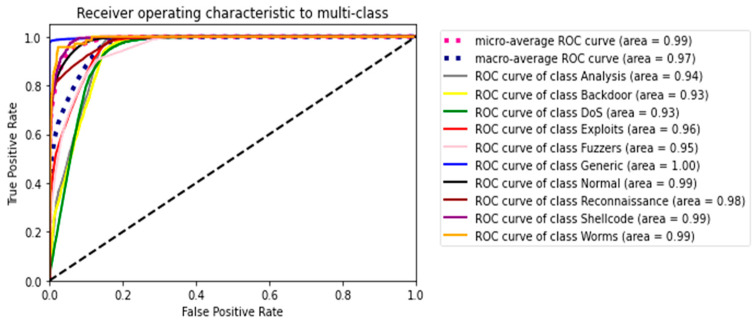
ROC-Curve for TPR vs. FPR of the proposed model for UNW-NB15.

The second experiment calculates different simulation results for OCNN-LSTM [1] and OCNN-LSTM with transfer learning (proposed model). Figure 9, Figure 10, Figure 11, Figure 12, Figure 13 and Figure 14 present the simulation results for Experiment 2 of the proposed model for the ToN-IoT dataset. Figure 9 presents the simulation results for the confusion matrix of the proposed model, Figure 10 presents the loss function evolution during training, Figure 11 presents the accuracy evolution during training, Figure 12 presents the precision evolution during training, and Figure 13 presents the recall curve for training of the proposed model for UNW-NB15. Figure 14 presents the ROC curve for TPR vs. FPR in the proposed model for UNW-NB15.

Table 6 presents the simulation results of the experiment of the proposed model and the existing OCNN-LSTM, CNN, and LSTM models for the UNW-NB15 dataset. The existing CNN-LSTM [1] model achieved a precision of 90.91%, recall of 51.66%, accuracy of 91.02%, and F1-score of 46.31%. We applied the transfer learning method with an optimized CNN and LSTM in the proposed model. The optimized CNN fine-tuned the hyperparameters, and the TL method enhanced the prediction accuracy by using the knowledge transformation.

The proposed model achieves a precision of 94.2%, recall of 55.01%, accuracy of 92.71%, and F1-score of 45.12%. This experimental analysis has proven that, after applying the TL method, the performance of the CNN-LSTM model is slightly increased. The proposed model presents better results for the UNW-NB-15 dataset.

## 5. Discussion and Conclusions

This research presented a hybrid intrusion detection model using OCNN-LSTM with transfer learning for IIoT networks in Industry 4.0. This research utilizes the popular IIoT datasets ToN-IoT and UNW-NB15. The proposed HIDM and existing CNN models were implemented on these two datasets. The proposed model uses transfer learning, which helps to enhance the prediction accuracy. The TL method transfers the training model knowledge into the OCNN-LSTM model, which allows for this model to more efficiently and precisely predict the attack classes from datasets. We applied the transfer learning method with an optimized CNN and LSTM in the proposed model. The optimized CNN fine-tuned the hyperparameters, and the TL method enhanced the prediction accuracy by using the knowledge transformation. The proposed HIDM model achieved a precision of 92.7% for the ToN-IoT dataset and a precision of 94.25%for the UNW-NB15 dataset, which proves the proposed model’s strength.

Memory and processing time are the two critical issues experienced when conducting this research, so we utilized class weights in place of scores, wherein the proposed HIDM was constrained by CPU capabilities such as storage, exaction memory, and testing and training time. We will overcome the training and validation loss in a future work. We will implement the proposed model in a real-time I4.0 environment to monitor and detect live data and will also try to compare it with more profound learning models.

## Figures and Tables

**Table 1:** Comparative analysis of the existing IDS research.

Reference	Model Used	Dataset	Outcome
[7]	Machine and deep learning algorithms	X-IIoTID	Deep learning methods achieved higher accuracy.
[8]	Federated learning and edge devices	X-IIoTID	Binary classification achieved 97.89% accuracy.
[9]	Deep neural network	ISOT, NSL-KDD, and X-IIoTID	DNN achieves high accuracy.
[10]	Clustering algorithm, expectation max, and ANN	UNSW-NB15 and KDD99	Expectation max achieves an accuracy of 81.25%.
[11]	GA-LR (Genetic algorithm with Logistic Regression)	UNSW-NB15	Accuracy of 89.87%.
[12]	Light gradient boosting machine	UNSW-NB15	Accuracy of 87.48%.
[13]	Variation of long short-term memory (VLSTM)	UNSW-NB15, ToN-IoT	Precision 98.58%.
[14]	Extreme learning machine (ELM)-based IDS	NSL-KDD and UNSW-NB15	Accuracy 91.87%.
[15]	Deep neural networks	UNSW-NB15, Kyoto, KDD-Cup99, CICIDS, NSL-KDD, and WSN-DS	The precision of 94.81%.
[16]	ANN for binary classification	UNSW-NB15	Accuracy 87.29%.
[17]	Two-stage TABU search (TS-TS) algorithm	UNSW-NB15	Precision 84.23%.
[18]	Xgboost with linear regression	UNSW-NB15 and ToN-IoT	Accuracy 79.59%.
[19]	LTSM with RNN	UNSW-NB15	Accuracy 88.31%.
[20]	Deep auto-encoder with LSTM	UNSW-NB15	Accuracy 91.81%.
[21]	Machine learning	ToN-IoT and X-IIoTID	Precision 87.84%.
Hybrid Model	Optimized CNN with transfer learning	UNW-NB15 and ToN-IoT	92.7%precision for ToN-IoT and 94.2% for the UNW-NB15 dataset.

**Table 2:** UNW-NB15 dataset descriptions.

Data Based on Events	Total Records
Normal	38,500
Back-door	950
Worms	174
Reconnaissance	978
Fuzzers	7500
DoS	4800
Shellcode	978
Exploits	12,440
Generic	18,554

**Table 3:** ToN-IoT dataset descriptions.

Data Based on Events	Total Records
Normal	79,638
Denial of service (DoS)	33,753
Back-door	50,811
Distributed denialofservice (*D*DoS)	61,650
MITM	105
Injections	45,265
Ransomware	7280
Scanning	71,401
Cross-site scripting (XSS)	21,089
Password	17,185

**Table 4:** The outcome of the proposed model for the TON-IoT dataset.

Model Type	Parameters	Source Type			
		Win-7	Win-10	Win-10 Network	Network type
OCNN-LSTM [1]	Training	23,789	29,878	98,9145	1,854,791
	Testing	6897	8178	325,147	5,478,985
	Epoch 50	30	47	4	2
OCNN-LSTM with Transfer Learning	Training	23,789	29,878	989,145	1,854,791
	Testing	6897	8178	325,147	5,478,985
	Epoch 50	34	45	6	3

**Table 5:** The outcome of the proposed model for the ToN-IoT dataset.

Model Type	Parameters	Class Type										
		Normal	DoS	DDoS	Back-Door	MITM	Injections	Ransomware	Scanning	Password	XSS	Average
Existing OCNN-LSTM [1]	Precision	1	0.78	0.86	0.89	0.89	0.89	0.89	0.88	0.92	0.91	0.891
	Recall	1	0.08	0.07	0.06	0.91	0.61	0.45	0.78	0.35	0.65	0.496
	Accuracy	1	0.85	0.87	0.71	0.91	0.81	0.89	0.88	0.94	0.87	0.873
	F1-Score	1	0.08	0.07	0.06	0.91	0.61	0.45	0.78	0.25	0.65	0.486
Existing CNN	Precision	1	0.71	0.79	0.85	0.81	0.812	0.82	0.802	0.812	0.80	0.81
	Recall	1	0.06	0.05	0.05	0.8	0.55	0.40	0.70	0.32	0.60	0.48
	Accuracy	1	0.81	0.76	0.63	0.81	0.71	0.79	0.78	0.74	0.77	0.73
	F1-Score	1	0.07	0.06	0.05	0.81	0.54	0.41	0.71	0.21	0.61	0.42
Existing LSTM	Precision	1	0.73	0.81	0.86	0.84	0.85	0.86	0.85	0.87	0.85	0.85
	Recall	1	0.07	0.06	0.06	0.85	0.59	0.41	0.70	0.31	0.62	0.47
	Accuracy	1	0.82	0.77	0.66	0.85	0.76	0.81	0.82	0.79	0.79	0.78
	F1-Score	1	0.071	0.062	0.050	0.82	0.52	0.40	0.71	0.21	0.60	0.44
Proposed OCNN-LSTM with Transfer Learning	Precision	1	0.91	0.9	0.925	0.932	0.918	0.91	0.89	0.93	0.95	0.927
	Recall	1	0.081	0.074	0.065	0.94	0.689	0.51	0.79	0.41	0.68	0.5239
	Accuracy	1	0.97	0.98	0.91	0.93	0.96	0.94	0.89	0.95	0.91	0.944
	F1-Score	1	0.091	0.087	0.098	0.94	0.78	0.56	0.87	0.45	0.78	0.566

**Table 6:** The outcome of the existing and proposed models for the UNW-NB15dataset.

Model Type	Parameters	Class Type									
		Normal	Back-Door	Worms	Reconnaissance	Fuzzers	DoS	ShellCode	Exploits	Generic	Average
Existing OCNN-LSTM [1]	Precision	1	0.87	0.89	0.91	0.92	0.91	0.901	0.867	0.914	0.909
	Recall	1	0.078	0.089	0.078	0.95	0.78	0.55	0.67	0.45	0.516
	Accuracy	1	0.89	0.87	0.91	0.9	0.87	0.914	0.89	0.95	0.910
	F1-Score	1	0.66	0.45	0.55	0.087	0.074	0.056	0.84	0.45	0.463
OCNN-LSTM with Transfer Learning	Precision	1	0.91	0.94	0.93	0.94	0.93	0.95	0.93	0.95	0.942
	Recall	1	0.085	0.0901	0.0845	0.96	0.812	0.65	0.712	0.556	0.550
	Accuracy	1	0.91	0.86	0.92	0.93	0.914	0.923	0.923	0.96	0.927
	F1-Score	1	0.067	0.54	0.65	0.086	0.087	0.066	0.89	0.67	0.451
Existing CNN	Precision	1	0.81	0.82	0.82	0.82	0.81	0.801	0.827	0.814	0.809
	Recall	1	0.068	0.079	0.068	0.85	0.68	0.45	0.57	0.41	0.456
	Accuracy	1	0.809	0.807	0.801	0.81	0.81	0.814	0.809	0.82	0.810
	F1-Score	1	0.566	0.40	0.45	0.077	0.070	0.046	0.74	0.35	0.413
Existing LSTM	Precision	1	0.84	0.83	0.84	0.83	0.83	0.821	0.847	0.824	0.829
	Recall	1	0.071	0.075	0.071	0.88	0.72	0.52	0.59	0.46	0.48
	Accuracy	1	0.829	0.827	0.831	0.831	0.841	0.854	0.839	0.852	0.850
	F1-Score	1	0.596	0.46	0.465	0.087	0.080	0.056	0.84	0.45	0.483

## Data Availability

Not Applicable.

## Abbreviations

The following abbreviations are utilized in this research.

**Abbreviation**	**Details**
DL	Deep learning
TL	Transfer learning
IDS	Intrusion detection system
CNN	Convolution neural network
LSTM	Long short-term memory
DoS	Denial of service attack
DDoS	Distributed denial of service attack
IoT	Internet of Things
IIoT	Industrial Internet of Things
OCNN	Optimized CNN
GWO	Grey wolf optimizer
HIDM	Hybrid intrusion detection model
H-M	Human-to-machine
M-M	Machine-to-machine
ML	Machine learning
ANN	Artificial neural network
GA	Genetic algorithm
SVM	Support vector machine
PSO	Particle swarm optimization
Light-GB	Light gradient boosting
IoD	Internet of Data
IoP	Internet of People
IoS	Internet of Services
